# Incremental cost-utility of sevelamer relative to calcium carbonate for treatment of hyperphosphatemia among pre-dialysis chronic kidney disease patients

**DOI:** 10.1186/s12882-016-0256-0

**Published:** 2016-04-28

**Authors:** Hai V. Nguyen, Saideep Bose, Eric Finkelstein

**Affiliations:** Program in Health Services and Systems Research, Duke-NUS Medical School, 8 College Road, Singapore, 169857 Singapore; Division of Cardiac, Thoracic, and Vascular Surgery, National University Hospital, 5 Lower Kent Ridge Road, Singapore, 119074 Singapore

**Keywords:** Sevelamer, Calcium carbonate, Dialysis, Hyperphosphatemia, Chronic kidney disease, Cost-effectiveness

## Abstract

**Background:**

Sevelamer is an alternative to calcium carbonate for the treatment of hyperphosphatemia among non-dialysis dependent patients with chronic kidney disease (CKD). Although some studies show that it may reduce mortality and delay the onset of dialysis when compared to calcium carbonate, it is also significantly more expensive. Prior studies looking at the incremental cost-effectiveness of sevelamer versus calcium carbonate in pre-dialysis patients are based on data from a single clinical trial. The goal of our study is to use a wider range of clinical data to achieve a more contemporary and robust cost-effectiveness analysis.

**Methods:**

We used a Markov model to estimate the lifetime costs and quality-adjusted life years (QALYs) gained for treatment with sevelamer versus calcium carbonate. The model simulated transitions among three health states (CKD not requiring dialysis, end-stage renal disease, and death). Data on transition probabilities and utilities were obtained from the published literature. Costs were calculated from a third party payer perspective and included medication, hospitalization, and dialysis. Sensitivity analyses were also run to encompass a wide range of assumptions about the dose, costs, and effectiveness of sevelamer.

**Results:**

Over a lifetime, the average cost per patient treated with sevelamer is S$180,724. The estimated cost for patients treated with calcium carbonate is S$152,988. A patient treated with sevelamer gains, on average, 6.34 QALYs relative to no treatment, whereas a patient taking calcium carbonate gains 5.81 QALYs. Therefore, sevelamer produces an incremental cost-effectiveness ratio (ICER) of S$51,756 per QALY gained relative to calcium carbonate.

**Conclusion:**

Based on established benchmarks for cost-effectiveness, sevelamer is cost effective relative to calcium carbonate for the treatment of hyperphosphatemia among patients with chronic kidney disease initially not on dialysis.

**Electronic supplementary material:**

The online version of this article (doi:10.1186/s12882-016-0256-0) contains supplementary material, which is available to authorized users.

## Background

Chronic kidney disease (CKD), defined as having two consecutive eGFR values <60 mL/min/1.73 m^2^ separated by at least 90 days, is on the rise in Singapore. The number of CKD patients increased more than 2-fold between 2007 and 2011, from 4734 to 10,245 cases. CKD is now the 9th leading cause of death in Singapore. It also imposes significant costs on the health care system. For example, more than 1000 CKD patients are now receiving dialysis, often at highly subsidized rates [1].

One of the primary causes of increased healthcare utilization and premature mortality among CKD patients is hyperphosphatemia. This occurs when serum phosphate levels are abnormally high, defined to be greater than 4.6 mg/dL in CKD patients and greater than 5.5 mg/dL in dialysis patients, according to the KDOQI guidelines [2]. Healthy individuals are able to rid the body of excess phosphate partly through urinary excretion. However, as renal function worsens, this mechanism is significantly impaired. This leads to increased vascular calcification and greater risk of cardiovascular events. Numerous studies have shown a dose/response relationship between hyperphosphatemia in CKD and increased cardiovascular diseases (CVD) and all-cause mortality [3, 4]. Standard treatment for hyperphosphatemia, in addition to consumption of a low phosphate diet, typically includes administration of calcium-based binders and non-calcium based binders that are able to block absorption of ingested phosphate. More recently, the prescription medication, sevelamer has been used as a non-calcium-based alternative. Studies have shown that sevelamer is at least as effective as calcium binders in reducing serum phosphate levels while causing less vascular calcification [5]. A recent randomized controlled trial of CKD patients not on dialysis [6] found that sevelamer delays progression to dialysis, and a meta-analysis concluded that sevelamer leads to improved survival outcomes [7].

Despite the health benefits, sevelamer is seven times as expensive as calcium carbonate in Singapore. This raises the question of whether sevelamer is a good use of scarce healthcare resources. To our knowledge, only two studies have looked at the incremental cost-effectiveness of sevelamer versus calcium carbonate in CKD patients; both relied on the clinical data from the INDEPENDENT trial [6]. The first study by Thompson et al. [8] used a Markov model with three health states (non-dialysis CKD, end-stage renal disease and all-cause death) to simulate the lifetime cost utility of sevelamer versus calcium carbonate for pre-dialysis CKD patients in the UK. They found that sevelamer has an incremental cost effectiveness ratio (ICER) of £23,878 (S$49,000) per QALY gained, which is considered cost-effective using the National Institute for Health and Care Excellence (NICE) benchmark of £30,000 (S$61,000) per QALY gained. However, Thompson et al. assumed a mortality hazard ratio of sevelamer compared to calcium carbonate of 0.45. This is well below the recent estimate from the Jamal et al. 2013 meta-analysis, which reports a mean hazard ratio of 0.88 [7]. Moreover, Thompson et al. assumed a constant dose for sevelamer (2.184 g/day) whereas CKD patients tend to increase to higher and more expensive doses over time, and especially after dialysis initiation. As a result of these assumptions, their cost-effectiveness ratio may be overly optimistic. The second study by Ruggeri et al. [9], also used an overly optimistic mortality hazard for sevelamer and limited the time-frame of analysis to three years, leaving the question about long term cost effectiveness unanswered.

Given the limitations of prior studies, the goal of this study is to revisit the long term cost-effectiveness of sevelamer relative to calcium carbonate. We create a Markov model using a specific mortality hazard from Jamal et al. and, consistent with current practice, specific higher doses of sevelamer that increase for a given patient over time. To the extent possible, we tailor the model for use in Singapore, the country of focus for this effort, but it can easily be applied to other countries.

## Methods

### Model structure

**Fig. 1:**
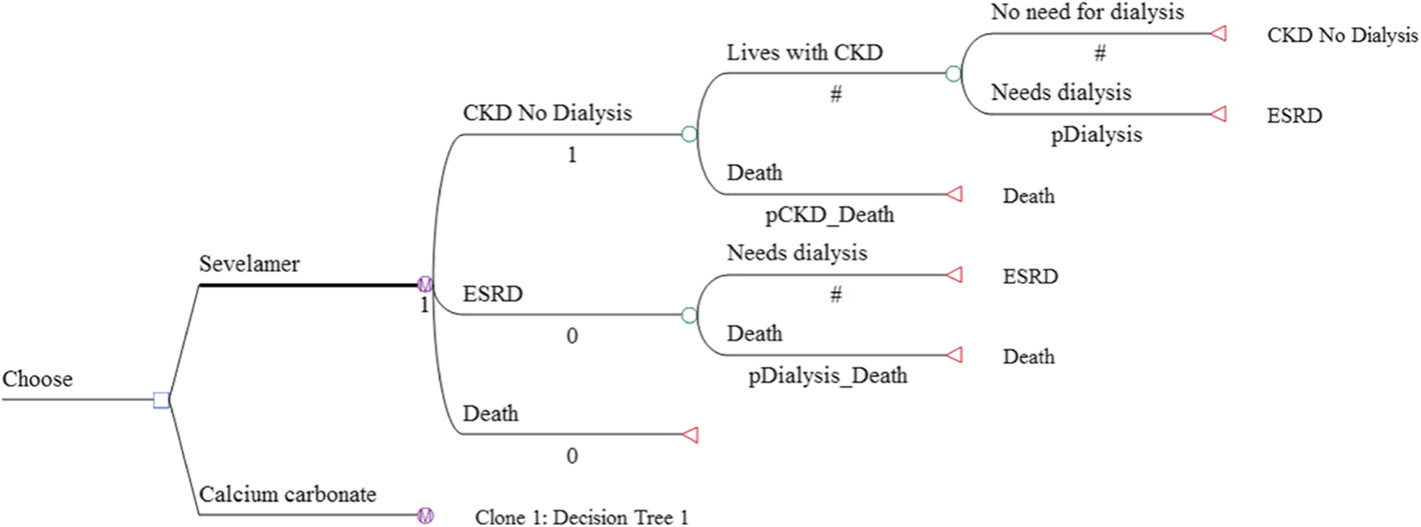
Markov decision tree

We developed a cohort Markov decision model using TreeAge Pro 2013 to simulate the ICER of sevelamer among incident CKD patients who have not yet started dialysis. The hypothetical cohort consisted of 1000 patients with mean age of 60. Age 60 corresponds to the average age of a representative cohort of CKD patients in Singapore [1]. As with Thompson et al., the Markov model considered 3 health states: CKD with no dialysis, end-stage renal disease, and all-cause mortality. Transplant was not included in the model because the number of transplants conducted in Singapore is very small (less than 60 cases per year). In any period, a patient in the ‘CKD with no dialysis’ state could either stay dialysis-free or transition to dialysis. Once dialysis is initiated, the patient was assumed to continue with dialysis until death, which may occur in any time period. Mortality risks are time-dependent and differ between non-dialysis CKD and end-stage renal disease patients. The base model was run for multiple one-year cycles until all cohort members died (i.e., lifetime horizon). The Markov decision tree for the two treatment drugs under comparison is presented in Fig. 1.

### Model inputs

The cost input data is Singapore specific. Data for other model inputs were obtained from the published literature and where possible, from studies that were specific to Singaporean or Asian populations. Model inputs are summarized in Table 1. No informed consent was needed as only secondary data was used in the analysis.

**Table 1:** Model inputs

Variable name	Base case	Range	Source
Markov basic parameters in the base case			
Time horizon	Lifetime (30 years)	2–40 years	Assumed
Cycle length	1 year	–	Assumed
Discount rate	3.5 %	–	NICE, 2013
Transitional probabilities			
Probability of dialysis initiation	Age dependent		Di Iorio et al. 2012 [6, 10]
Mortality risk for CKD patients without dialysis	Age dependent		Calculated based on data from literature
Mortality for end-stage renal disease patients	Age dependent		Calculated based on data from literature
Treatment parameters			
Hazard ratio, sevelamer versus calcium carbonate	0.88	0–1	Jamal et al., 2013 [7]
Hazard ratio, CKD versus no CKD	1.83	0.915–2.745	Di Iorio et al. 2012 [6, 10]
Costs			
Drug acquisition unit price			
Sevelamer (S$/g)	1.41	0–4.23	SANOFI
Calcium carbonate (S$/g)	0.192	0.06–0.25	SANOFI
Usage dose			
For non-dialysis CKD patients			
Sevelamer (g/day)	2.184		Thompson et al. 2013 [8]
Calcium carbonate (g/day)	2.950	0–6	Thompson et al. 2013 [8]
For end-stage renal disease patients			
Sevelamer (g/day)			
Year 1	2.4		Assumed
Years 2–4	4.8		Assumed
Years 5–7	7.2		Assumed
Years 8–10	9.6		Assumed
Calcium carbonate (g/day)	2.95	0–6	Thompson et al. 2013 [8]
Hospitalization costs			
Hospitalization cost per day (S$)	444		Singapore MOH
Length of hospitalization for dialysis patients with sevelamer (days)	12.3	5–20	St Peter et al. 2008 [19]
Length of hospitalization for dialysis patients with calcium carbonate (days)	13.9	5–20	St Peter et al. 2008 [19]
Length of pre-dialysis hospital stay for patients with sevelamer (days per hospitalization)	5.8		Khan et al. 2002 [18]
Length of pre-dialysis hospital stay for patients with calcium carbonate (days per hospitalization)	6.6		Khan et al. 2002 [18]
Annual risk of pre-dialysis hospitalization	0.58		Go et al. 2004 [17]
Dialysis costs			
Haemodialysis cost (S$/month)	2517		Singapore MOH
Peritoneal dialysis costs (S$/month)	1670		Singapore MOH
Proportion of patients with haemodialysis dialysis in Singapore (%)	82.3		Singapore MOH
Total dialysis costs (per year)	28,400	14,200–42,600	
Utilities			
CKD patients not on dialysis	0.85	0.8–0.90	Gorodetskaya et al. 2005 [20]
End-stage renal disease patients	0.72	0.65–0.8	Gorodetskaya et al. 2005 [20]

All costs are in Singapore Dollars (S$)

### Transition probabilities

Transition probabilities were obtained from published studies and from our own calculations. The probabilities for transitioning to dialysis in each cycle were obtained from Di Iorio et al., which presents results of a three year randomized controlled trial designed to test the clinical effectiveness of sevelamer versus calcium carbonate for treatment of hyperphosphatemia among CKD patients in Italy [6].

Although Di Iorio et al. also contains mortality transition probabilities, this data is only available for the three-year duration of the clinical trial. Further, in an erratum it was clarified that patients were right censored (i.e., removed) from analysis when they entered dialysis [10]. As a result, the mortality data from their paper is not an accurate representation of the lifetime mortality hazard. Therefore, we estimated mortality transition probabilities based on data from the literature. To estimate mortality risks of non-dialysis CKD patients, we used data on three parameters: (a) age-specific mortality risks from Singapore life-tables [11], (b) mortality hazard ratios for CKD patients relative to non-CKD patients based on a large Taiwanese cohort study [12], and (c) mortality hazard ratios for sevelamer relative to calcium carbonate among CKD patients obtained from a recent meta-analysis [7].

Specifically, we first inflated the age specific mortality risks from the Singapore life tables by the hazard ratio for CKD patients. These inflated mortality risks were assumed to be the mortality risks for CKD patients treated with calcium carbonate, which is currently standard treatment in Taiwan. We next calculated mortality risks for those treated with sevelamer by multiplying the mortality risks for CKD patients treated with calcium carbonate by the sevelamer-specific hazard ratio from the Jamal et al. study. For mortality risks for CKD patients on dialysis, we used age-specific mortality risks from the US Renal Data System [13] as no similar data was available for Singapore but adjusted them down by 25 %. This adjustment factor is based on Wong et al. [14] showing that the mortality risk of Asian American end-stage renal disease patients was 0.75 compared to that of non-Asian American end-stage renal disease patients. These mortality risks were then differentiated between the two drugs using the same sevelamer-specific hazard ratio as above.

### Costs

Costs consist of three components: medication, dialysis, and hospitalizations. Annual drug costs for sevelamer and calcium carbonate were calculated as the product of the market acquisition unit price for each drug times its dosage per day times 365 days per year. Data on the acquisition prices (S$1.41/g for sevelamer and S$0.192/g for calcium carbonate) were provided by the manufacturer.

Dosage for each drug differs between non-dialysis CKD and end-stage renal disease patients. For non-dialysis CKD patients, constant doses of (2.184 g/day for sevelamer and 2.95 g/day for calcium carbonate) were assumed. For end-stage renal disease patients, higher doses over time are often required as the disease progresses. Therefore, dosage of sevelamer for these patients was set to be 2.4 g/day in the first year, 4.8 g/day in years 2–4, 7.2 g/day in years 5–7, and 9.6 g/day from year 8 onwards [15]. Due to the lack of data on changes in dosage over time for patients on calcium carbonate and because of its very low cost relative to sevelamer, we assumed a constant dose (2.95 g/day).

Costs for dialysis were calculated as the weighted average of the unsubsidized costs of haemodialysis and peritoneal dialysis made public by the Singapore Ministry of Health. The weights were the proportions of CKD patients in each dialysis modality (17.7 % for peritoneal and 82.3 % for haemodialysis), which were extracted from the Singapore Renal Registry Annual 2013 Report [1].

Hospitalization costs in pre-dialysis and dialysis phases were obtained by multiplying the non-subsidized hospitalization cost per day (S$445) provided by Singapore Ministry of Health [16] with the average number of hospitalized days per year for CKD patients and ESRD patients, respectively. The average number of hospitalized days per year for CKD patients was computed by multiplying the annual risk of hospitalization for CKD patients [17] with the average length of stay per hospitalization (6.6 days and 5.8 days for calcium carbonate and sevelamer patients, respectively [18]). The average number of hospitalized days per year for dialysis patients were estimated in St Peter et al., i.e., 12.3 inpatient days for dialysis patients who were treated with sevelamer versus 13.8 days for those on calcium carbonate [19].

### Health utility weights

Health utility weights were based on a time-trade-off analysis from Gorodetskaya et al. Specifically, the health utilities for a CKD patient undergoing or not undergoing dialysis are 0.72 and 0.85, respectively [20].

### Other model inputs

The age distribution of the cohort was defined to match that from the Singapore Renal Registry Annual Report 2013 [1]. As age is a significant risk factor for mortality of CKD patients, this real world age composition enables the model to track the disease progression for patients treated with sevelamer and calcium carbonate in Singapore more accurately than the conventional approach of assuming all cohort members start at the same age. Both future costs and utilities were discounted at 3.5 % as recommended by the UK NICE [21].

### Sensitivity analysis

We supplemented the base case analysis with both deterministic and probabilistic sensitivity analyses to examine the robustness of the results. In the one-way sensitivity analysis, we allowed each parameter to change in a range that is based on the literature (e.g., for drug dose) or is reasonably large (e.g., +/−50 % for hazard ratio and dialysis costs and +/-100 % for drug costs). Results are presented in terms of a Tornado diagram, with key variables shown in additional one way sensitivity charts. We also conducted a sensitivity analysis using a discount of 1.5 %.

In probabilistic sensitivity analysis, we assigned distributions to all input parameters and performed 10,000 Monte Carlo simulations where each simulation generated an ICER. Results are presented graphically as cost effectiveness acceptability curves which show the probability that each drug is cost-effective for any given cost-effectiveness threshold. We assumed beta distributions for all probabilities and utilities whose values were bounded between 0 and 1, normal distributions for hazard ratios and drug doses, and gamma distributions for medication and dialysis costs (to capture its non-negative and skewed features). The moments for these distributions were based on the point estimates and on our choice of a relatively large standard deviation (i.e., 25 % of the mean) [22].

## Results

The age distribution of our simulated cohort is displayed in Table 2. The average age of the cohort is 60, with patients aged between 50 and 70 accounting for more than two-third of the cohort (i.e., 71 %). All cohort members died after the model was run for 30 years.

**Table 2:** Initial age distribution of the cohort

Age	Percentage
0	0
20	1.2
30	3.5
40	8.4
50	21
60	24.9
70	24.9
80	15.7

Source: Singapore Renal Registry Annual Report 2013

The base case cost utility results are presented in Table 3. Over a lifetime, a patient treated with sevelamer costs more than a patient treated with calcium carbonate (S$180,724 versus S$152,988) but also gains more QALYs (6.34 versus 5.81). The ICER of sevelamer relative to calcium carbonate is S$51,756 per QALY gained. Compared with the NICE cost-effectiveness threshold of £30,000 or S$61,000 per QALY (based on the £ SGD exchange rate as of Nov. 24, 2012), sevelamer is cost effective compared with calcium carbonate.

**Table 3:** Base case incremental cost utility results (lifetime horizon)

Strategy	Cost (S$)	Incremental cost (S$)	QALYs	Incremental QALYs	ICER (S$/QALY)
Calcium carbonate	152,988	0	5.81	0	0
Sevelamer	180,724	27,735.6	6.34	0.5359	51,756

All costs are in Singapore Dollars (S$). *ICER* incremental cost-effectiveness ratio. Although our study cohort all starts out not requiring dialysis, a large majority of them progress to end-stage renal disease and require dialysis by the end of the model simulation

**Fig. 2:**
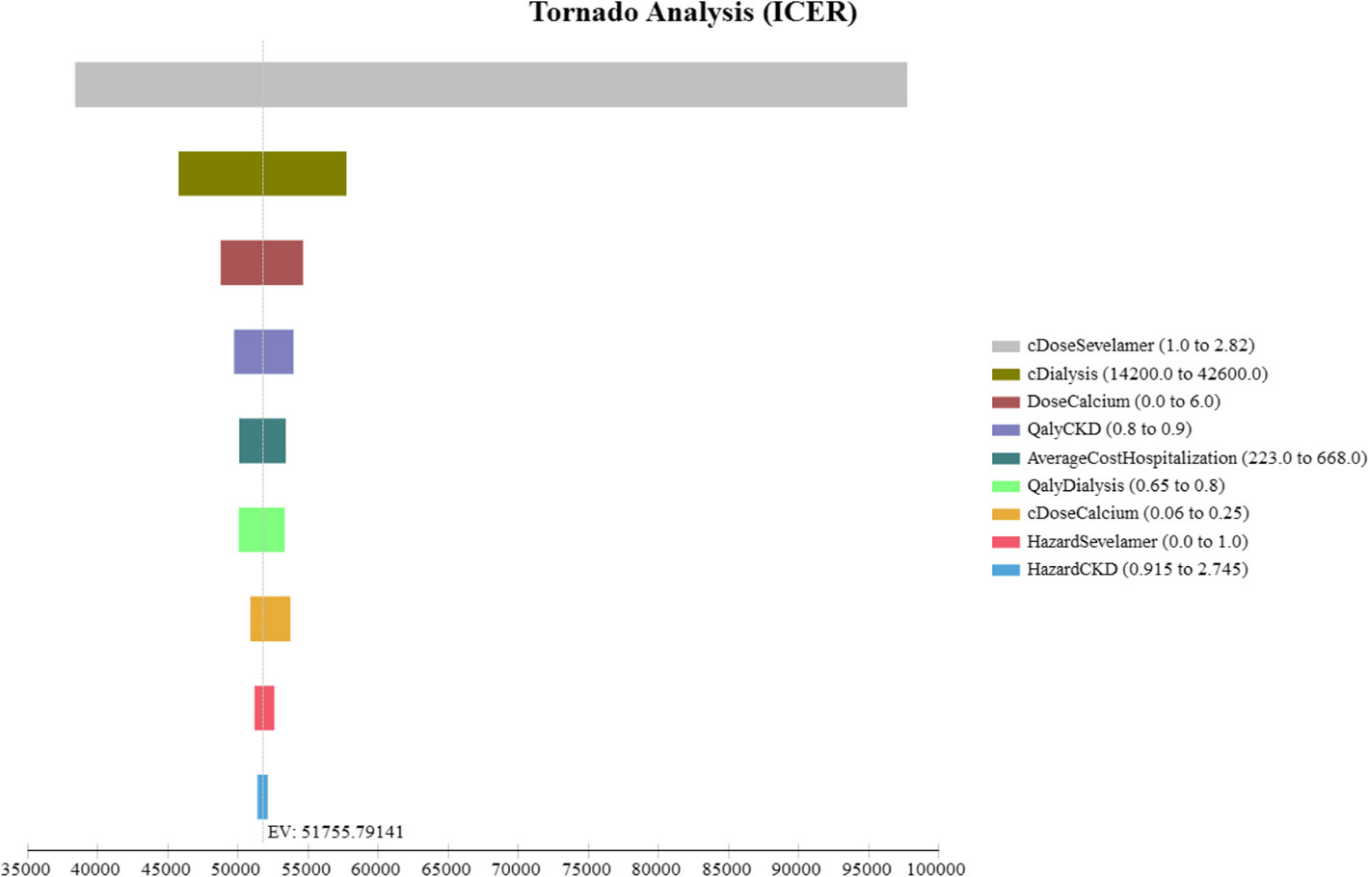
Tornado diagram

**Fig. 3:**
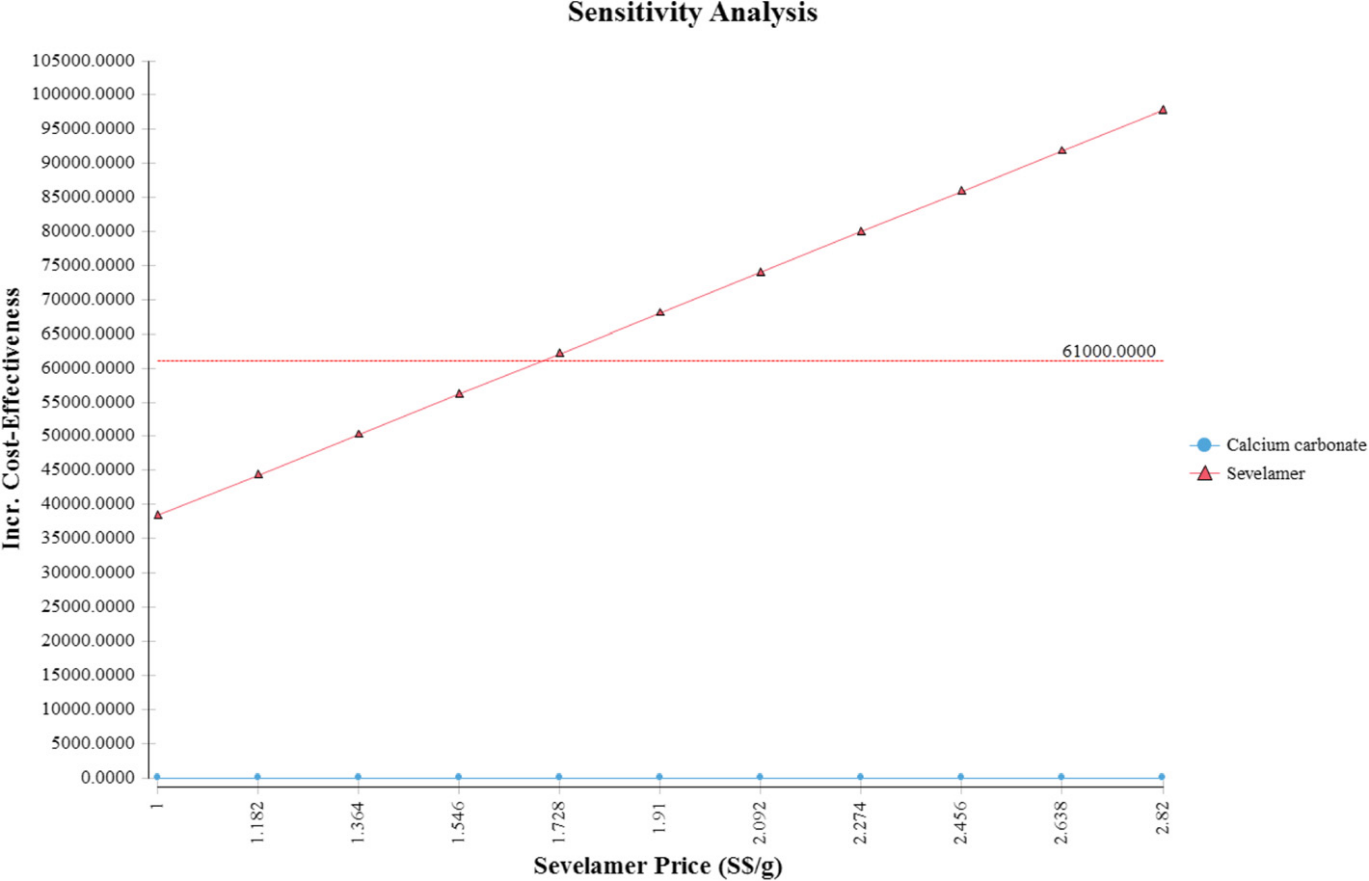
One-way sensitivity analysis (price of sevelamer)

The Tornado diagram, displayed in Fig. 2, shows that the ICER is most sensitive to changes in the prices of sevelamer and dialysis. Figure 3 shows the ICER for sevelamer as a function of its price. As expected, the ICER increases as sevelamer becomes more expensive. In particular, if the price of sevelamer exceeds S$1.69/g (the intersection point between the ICER line and the cost effectiveness threshold line), sevelamer is no longer cost effective. We also considered a high dose (i.e., three times the base-case dose) [23] and a low dose (i.e., 50 % of the base-case dose) for calcium carbonate. In both cases, sevelamer is still cost effective relative to calcium carbonate with ICER being S$45,986/QALY and S$53,198/QALY, respectively. Another sensitivity analysis indicates that sevelamer dominates calcium carbonate (i.e., has lower cost and greater effectiveness) when the time horizon is 6 years or less. Beyond six years, sevelamer remains cost effective, although it becomes less attractive as the time horizon expands. The reason for this is that, beyond six years, as the duration on treatment increases, both dialysis and sevelamer costs increase (and at the higher dosage) at a faster rate than the increase in QALYs.

**Fig. 4:**
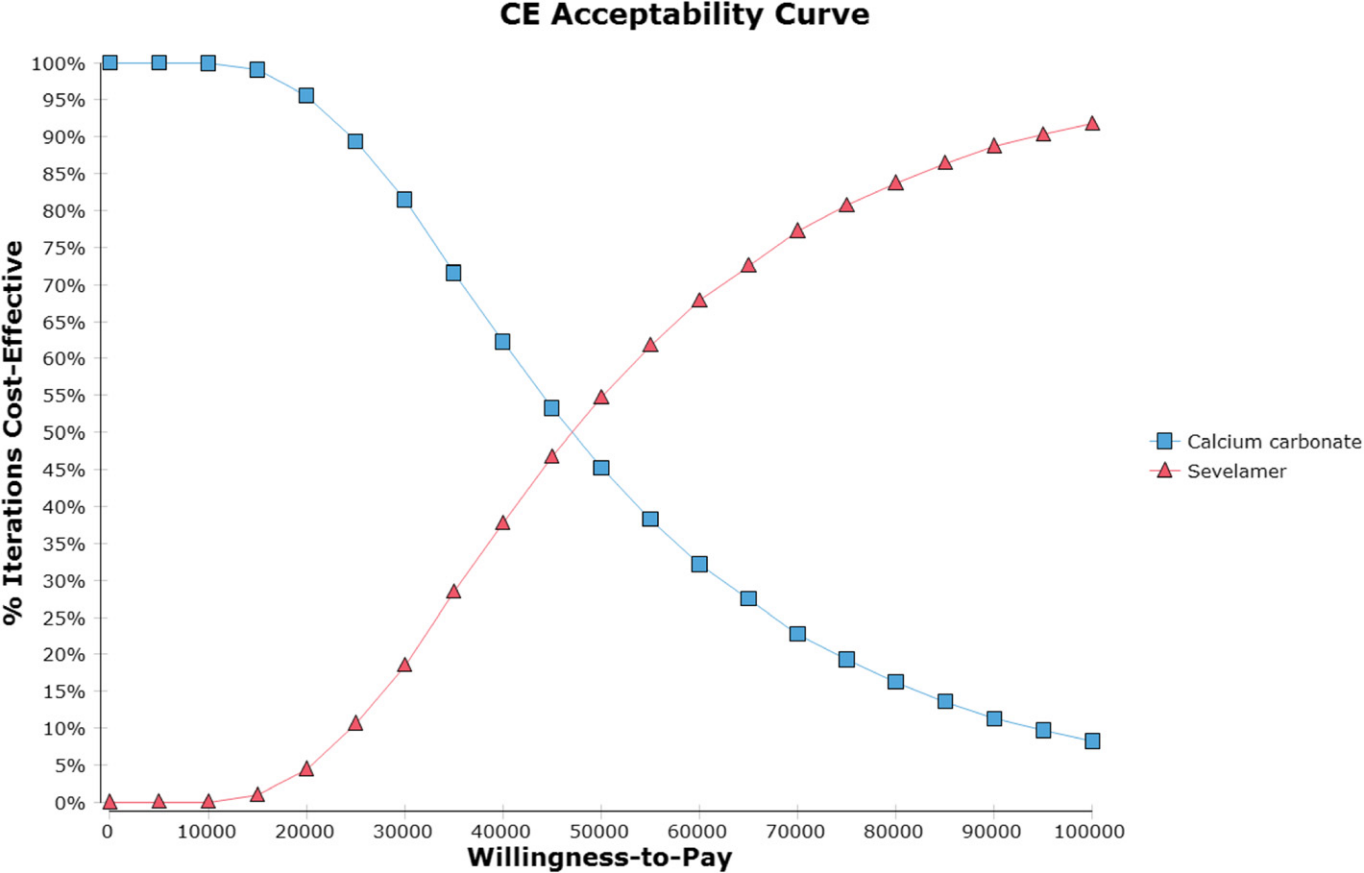
Cost-effectiveness acceptability curve

Figure 4 displays the cost effectiveness acceptability curves for sevelamer and calcium carbonate derived from the probabilistic sensitivity analysis around the base case assumptions. If the willingness to pay (WTP) per QALY gained is S$61,000 (i.e., the NICE threshold), sevelamer is cost effective in 69 % of iterations.

## Discussion

This study presents results of a cost utility analysis comparing sevelamer with calcium carbonate as alternative treatments for hyperphosphatemia among CKD patients. The model was updated with the best available data and tailored for use in Singapore. Base case results suggest that, using common thresholds for cost-effectiveness, sevelamer is cost effective with an ICER of S$51,756 per QALY gained. The results are robust to reasonable variations in a number of key parameters.

**Fig. 5:**
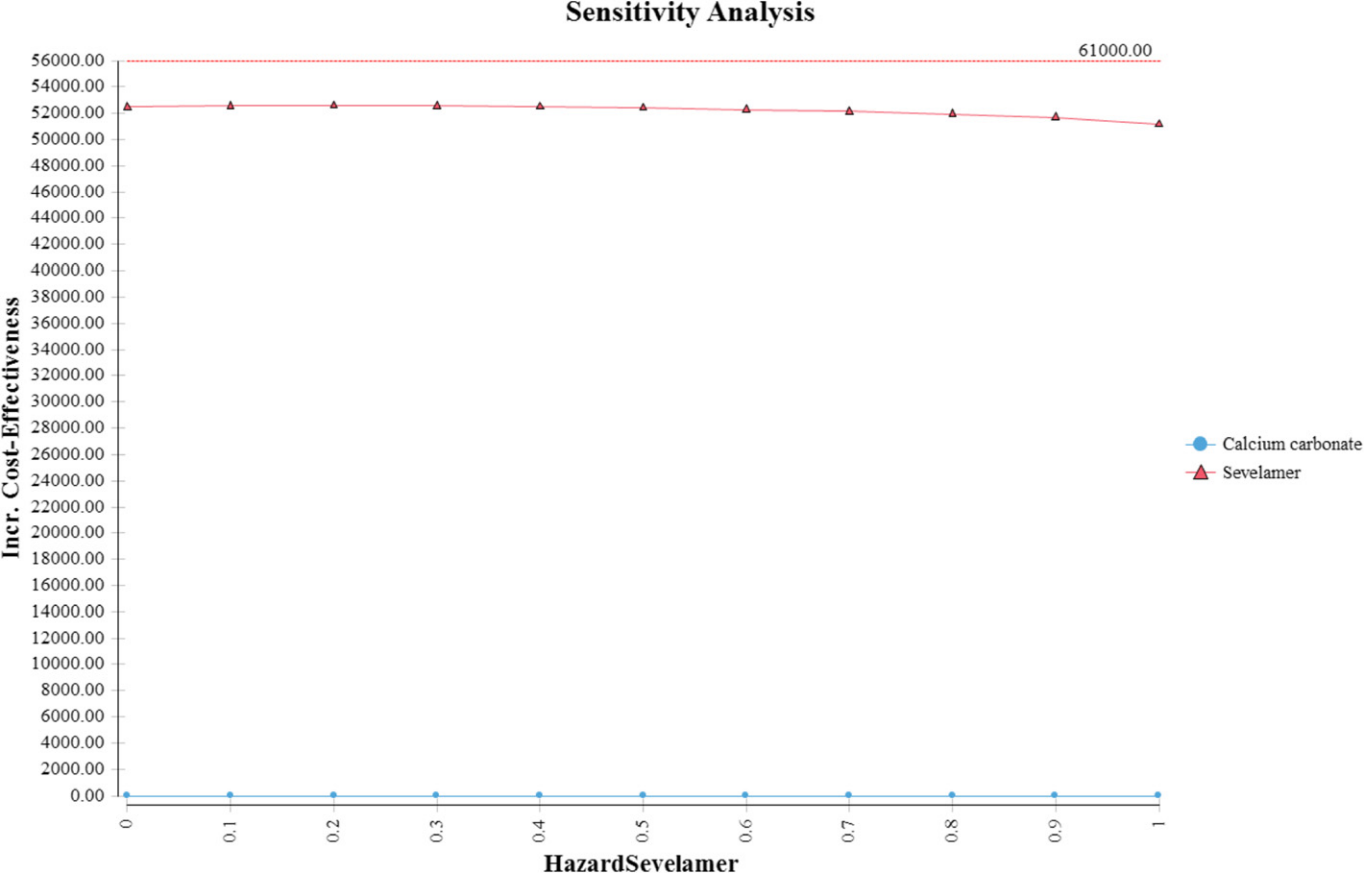
Relationship between incremental cost-effectiveness ration and hazard ratio for sevelamer

Thompson et al. generated an ICER of sevelamer relative to carbonate calcium of £23,878 (S$49,000) per QALY gained [8]. Ironically, their slightly higher ICER is due to use of a *lower* mortality hazard ratio (i.e., 0.45). As shown in Fig. 5, sevelamer becomes *less* cost effective as it becomes *more* effective at reducing mortality risks. This is because a more effective sevelamer means that more patients are on dialysis and for longer time periods. This increases dialysis and sevelamer costs (and at higher dosages) at a greater rate than the increase in QALYs, making sevelamer less cost effective. When using the same hazard ratio as Thompson et al., our ICER increases to S$52,477 per QALY, which is similar to their estimate.

Ruggeri et al. found that sevelamer dominates calcium carbonate (i.e., more effective and less expensive) when assuming an optimistic mortality hazard ratio for sevelamer and a much shorter time horizon (i.e., three years) [9]. When we apply their mortality hazard and a three-year time horizon, our model generates an analogous result. The reason for the more favorable cost effectiveness result in the short term is that when the time horizon is short, most patients are in the no-dialysis state. Consequently, a more effective sevelamer (i.e., lower mortality hazard ratio) delays the onset and costs of dialysis, making sevelamer more cost effective. In the longer term, patients transition to dialysis, which results in much higher costs and a lower cost-effectiveness ratio.

Our study has a number of limitations. The first limitation, given our focus on Singapore, is the lack of Singapore specific data for a number of model inputs. With the exception of costs, the base case age distribution and the mortality hazard ratio, other parameters were not specific to Singapore. In particular, we assumed the health utilities for CKD patients do not differ between Caucasians and Singaporeans. Given access to treatment is similar in the US (where the study was conducted) and Singapore, we see no reason to believe that this assumption would not hold. Moreover, we allowed this and other key variables to change in a reasonably large range in the sensitivity analyses, and the primary conclusion remains unchanged.

Another limitation is that we assumed the mortality risks for the CKD subgroups could be estimated solely with information on overall mortality rates, mortality hazard rates for CKD patients and rates for sevelamer-treated CKD patients. In reality, these calculations should be based on both hazard rates and the prevalence of each subgroup in the population. However, no data on age-specific prevalence for CKD patients (regardless of treatment regimen) is available for Singapore. Consequently, our calculation of CKD mortality risks did not use the prevalence of the CKD subgroup. In doing so, we essentially assumed that the mortality risks for the general population are equal to the mortality risks for the non-CKD population. However, because CKD patients represent a small fraction of the total population, this should have a negligible effect on the overall and sevelamer specific CKD mortality rates and therefore on the estimated ICERs.

A further limitation of the paper is that it compared the outcomes of sevelamer to calcium carbonate. In certain countries, such as the United States only calcium acetate is approved for non-dialysis CKD patients. However, in other countries such as Singapore, there is currently no such restriction. Furthermore, the most recent meta-analysis on the topic by Jamal et al., which was one of the data sources for our study, grouped calcium carbonate and calcium acetate into one category. Finally, our analysis only modelled the scenario where a patient received one phosphate binder. In clinical practice some patients may require multiple binders for adequate phosphorus control.

## Conclusions

From a third party payer perspective and considering a lifetime time horizon, sevelamer is likely to be cost effective relative to calcium carbonate as a treatment for hyperphosphatemia in CKD patients in Singapore and in countries with similar health systems.

### Ethics approval and consent to participate

Not applicable.

### Consent to publish

Not applicable.

### Availability of data and materials

Source for data used in the analysis has been provided in Additional file 1: Table S1.

## Appendix

**Table 4:** Incremental cost utility results (lifetime horizon) with discount rate of 1.5 %

Strategy	Cost (S$)	Incremental cost (S$)	QALYs	Incremental QALYs	ICER (S$/QALY)
Calcium carbonate	176,851	0	6.40	0	0
Sevelamer	212,103	35,252	7.05	0.65	53,848

All costs are in Singapore Dollars (S$). *ICER* incremental cost-effectiveness ratio. Although our study cohort all starts out not requiring dialysis, a large majority of them progress to end-stage renal disease and require dialysis by the end of the model simulation

## Additional file

Additional file 1: CHEERS checklist—Items to include when reporting economic evaluations of health interventions. (DOCX 19 kb)

## Abbreviations

CKD: chronic kidney disease
CVD: cardiovascular disease
ICER: incremental cost effectiveness ratio
NICE: National Institute for Health and Care Excellence
QALY: quality adjusted life years
WTP: willingness-to-pay
